# Benefit of Tetrahydrocannabinol versus Cannabidiol for Common Palliative Care Symptoms

**DOI:** 10.1089/jpm.2018.0658

**Published:** 2019-09-30

**Authors:** David J. Casarett, Jessica N. Beliveau, Michelle S. Arbus

**Affiliations:** ^1^ Department of Medicine at Duke University, Durham, North Carolina.; ^2^ Research Department, Strainprint Technologies Ltd., Toronto, Ontario, Canada.

**Keywords:** cannabis, palliative care, symptom control

## Abstract

***Objectives:*** To determine the relative contributions of tetrahydrocannabinol (THC) and cannabidiol (CBD) to patients' self-ratings of efficacy for common palliative care symptoms.

***Design:*** This is an electronic record-based retrospective cohort study. Model development used logistic regression with bootstrapped confidence intervals (CIs), with standard errors clustered to account for multiple observations by each patient.

***Setting:*** This is a national Canadian patient portal.

***Participants:*** A total of 2,431 patients participated.

***Main Outcome Measures:*** Self-ratings of efficacy of cannabis, defined as a three-point reduction in neuropathic pain, anorexia, anxiety symptoms, depressive symptoms, insomnia, and post-traumatic flashbacks.

***Results:*** We included 26,150 observations between October 1, 2017 and November 28, 2018. Of the six symptoms, response was associated with increased THC:CBD ratio for neuropathic pain (odds ratio [OR]: 3.58; 95% CI: 1.32–9.68; *p* = 0.012), insomnia (OR: 2.93; 95% CI: 1.75–4.91; *p* < 0.001), and depressive symptoms (OR: 1.63; 95% CI: 1.07–2.49; *p* = 0.022). Increased THC:CBD ratio was not associated with a greater response of post-traumatic stress disorder (PTSD)-related flashbacks (OR: 1.43; 95% CI: 0.60–3.41; *p* = 0.415) or anorexia (OR: 1.61; 95% CI: 0.70–3.73; *p* = 0.265). The response for anxiety symptoms was not significant (OR: 1.13; 95% CI: 0.77–1.64; *p* = 0.53), but showed an inverted U-shaped curve, with maximal benefit at a 1:1 ratio (50% THC).

***Conclusions:*** These preliminary results offer a unique view of real-world medical cannabis use and identify several areas for future research.

## Introduction

The past 10 years have seen growing enthusiasm for medical cannabis in the United States, Canada, and in Europe, especially among patients with serious life-threatening conditions.^1,2^ That enthusiasm has been coupled with growing evidence to support the benefits for medical cannabis for a variety of symptoms. Recent reviews and meta-analyses have pointed to benefits for pain, nausea, anorexia, spasticity, and several other symptoms.^3–8^

However, there is inadequate evidence to guide clinicians and patients in choosing the optimal ratio of the two most common cannabinoids, tetrahydrocannabinol (THC) and cannabidiol (CBD). These molecules have different receptors, physiological effects, and potential medical benefits. In particular, THC is associated with psychoactive effects such as euphoria, anxiety symptoms, paranoia, and hallucinations, whereas CBD is not.

Data that define the relative contributions of THC and CBD to symptom management could be very useful in suggesting an optimal ratio for a given symptom. Specifically, such data could help patients and clinicians to identify those symptoms for which THC offers the greatest contribution. That, in turn, would make it possible to use higher THC:CBD ratios only for those symptoms that are most THC responsive, avoiding unnecessary toxicity.

Ideally, optimal THC:CBD ratios would be defined in randomized controlled trials that evaluate effectiveness and toxicity of a range of ratios. However, such studies are difficult, time consuming, and expensive, particularly because in the United States cannabis is a schedule I substance. Therefore, the goal of this retrospective cohort study was to use patient reports to explore the optimal percentage of THC on the impact of common palliative care symptoms, and to gather preliminary data that can guide the design of future randomized controlled trials.

## Methods

### Setting and sample

Data were sourced from the Strainprint™ mobile app, which is a medical outcomes tracker providing medical cannabis users a means of tracking changes in symptoms as a function of different doses and types of cannabis. Strainprint allows patients to record and visualize their own cannabis use, including the symptom they are treating, strain name, licensed producer, dose, method of ingestion against prepopulated lab-verified products. The app provides patients with a summary of their own history, as well as recommendations based on the experiences of patients with the same symptoms. Strainprint uses a system of redeemable loyalty rewards to attract and retain a community of engaged patients, and to encourage participation. The system is monitored in real time with machine learning and artificial intelligence to monitor, prevent, and cleanse points gaming.

Patients open the app before cannabis use and use a drop-down menu to select the symptom they wish to treat. They are prompted to record their symptom severity on an 11-point numeric rating scale (0–10). Next, patients are asked to select the cannabis product they are using, route of administration, and dose. Finally, after use, patients are prompted to complete a second severity rating.

Data elements for this study were defined *a priori* and then extracted from the Strainprint database, stripped of identifiers to create a Health Insurance Portability and Accountability Act (HIPAA)-compliant limited dataset before transfer to Duke University for analysis.

We restricted the sample to instances of use for six symptoms: neuropathic pain, insomnia, anxiety symptoms, depressive symptoms, anorexia, and post-traumatic stress disorder (PTSD)-related flashbacks. If a patient also used cannabis for other symptoms, we included only their effectiveness ratings for the six symptoms mentioned. When patients used cannabis for more than one symptom at a time, they provided pre- and postratings for each.

We restricted the sample to only those products whose content was verified by laboratory testing. We also limited the sample to uses that were at least one hour apart to avoid inadvertent duplicated entries, and contamination of one effectiveness rating by another use. Finally, we restricted the sample to vaporizers only. Vaporizers are designed to deliver a standardized dose, unlike smoking, which can be influenced by rolling characteristics and user technique. Similarly, other routes of administration (e.g., oral and submucosal) are subject to variable delays in absorption and differences in metabolism, which would make comparisons challenging.

### Data analysis

First, we generated descriptive statistics to define unique patients, use patterns, timing, and reason for use. Next, we calculated the percentage THC in each product by dividing the THC content by the sum of THC and CBD content. This is not the true percentage THC by weight, but rather an expression of the THC:CBD ratio, expressed on a 0–100 scale. For example, a concentration that included 10% THC content would include 90% CBD content. We did not include other cannabinoids such as cannabinol, but those compounds are present in small amounts (generally <1% total) in most products.

We defined effectiveness as at least a three-point pre/post difference on patients' ratings of symptom severity before and after use. Although this threshold is arguably arbitrary, it has been used in other studies of the role of cannabis in managing symptoms, and especially pain.^9,10^ In this sample, a three-point difference corresponds to ∼1.5 standardized difference (1½ standard deviations) for the symptoms described here. We used case-wise deletion of uses when one or more data elements were missing.

For each symptom, we created a logistic regression model in which symptom reduction of at least three points was the outcome of interest. Each regression model included age, gender, and number of inhalations in each use. Models were clustered by user id to account for multiple uses per individual. For each model of the association of THC concentration and outcome, we calculated an odds ratio (OR) as a measure of effect size. Finally, we used the same models to calculate effectiveness (at least a 3-point decrease), adjusted for model covariates and stratified by THC percentage in 10-point increments.

This study was determined to be exempt by the Duke University Institutional Review Board. Stata statistical software (Stata MP2 11.0 for Mac; Stata Co, College Station, TX) was used for all statistical analysis.

## Results

Between October 1, 2017 and November 28, 2018, patients submitted a total of 895,512 uses. Of these, 659,561 (73.6%) uses were laboratory verified, and 308,878 of these were at least one hour apart. Of these, 107,999 uses were for a vaporizer. Finally, we restricted the sample to those with complete data (99,776) and then to at least one of the six selected symptoms (*n* = 2431 patients; 26,180 observations). The characteristics of the sample are described in Table 1.

**Table 1.** Patient Characteristics

*Symptom*	n	*Gender; male* n *(%)*	*Age mean (range)*	*Number uses*	*Preuse severity*	*Postuse severity*	*Improvement (%)*	*% THC*
Neuropathic pain	304	111 (36.6)	39 (18–86)	3391	6.14	3.45	42	71% (3–100%)
Anorexia	289	123 (42.9)	30 (18–69)	1664	6.43	2.28	77	94% (3–100%)
PTSD-related flashbacks	148	58 (39.7)	36 (18–78)	1144	6.02	2.23	78	91% (3–100%)
Insomnia	869	375 (43.2)	34 (18–76)	4613	7.19	3.18	71	95% (0–100%)
Anxiety symptoms	1086	466 (43.0)	33 (18–82)	9340	5.85	2.21	66	84% (1–100%)
Depressive symptoms	775	338 (43.7)	33 (18–78)	6028	5.90	2.70	61	90% (0–100%)

PTSD, post-traumatic stress disorder; THC, tetrahydrocannabinol.

Preuse symptom severity varied from 5.90 (depressive symptoms) to 7.19 (insomnia) and postuse severity ranged from 2.21 (anxiety symptoms) to 3.45 (neuropathic pain). The smallest reductions were seen for neuropathic pain (42.2% had at least a three-point reduction) and the largest for PTSD-related flashbacks (78.1%) (Table 1). Across symptoms, THC percentage ranged from 70.5% (neuropathic pain) to 94.9% (insomnia), and ranged from ∼0% to 100%.

Responses to vaporized cannabis, adjusted for THC:CBD ratio, dose, age, and gender, varied widely. The lowest adjusted effectiveness ratings were seen with neuropathic pain (47.0%; 95% confidence interval [CI]: 35.3–59.0) and depressive symptoms (61.4%, 95% CI: 56.0–66.5). Effectiveness for anxiety symptoms was slightly higher (66.0%, 95% CI: 60.6–71.0). The highest ratings of effectiveness were for anorexia (78.0%; 95% CI: 68.7–85.2), PTSD-related flashbacks (78.6%; 95% CI: 65.0–88.0), and insomnia (70.9%, 95% CI: 67.4–74.1).

Next, we evaluated each symptom separately and examined the association between THC:CBD ratio (expressed as percentage THC) and a three-point symptom improvement, adjusting for age, gender, and dose (Table 2). Several symptoms were very sensitive to increasing THC:CBD ratios. For instance, response increased with increasing THC:CBD ratio for neuropathic pain (OR: 3.58; 95% CI: 1.32–9.68; *p* = 0.012) and insomnia (OR: 2.93; 95% CI: 1.75–4.91; *p* < 0.001). There was a smaller positive association for depressive symptoms (OR: 1.63; 95% CI: 1.07–2.49; *p* = 0.022).

**Table 2.** Associations between Tetrahydrocannabinol Concentration (Tetrahydrocannabinol/Tetrahydrocannabinol+Cannabidiol) and Response (Three-Point Improvement)

*Symptom*	*Odds ratio (95% CI)*	*95% CI*	p
Neuropathic pain	3.58	1.33–9.68	0.012
Anorexia	1.61	0.70–3.73	0.265
PTSD-related flashbacks	1.43	0.60–3.41	0.415
Insomnia	2.93	1.75–4.91	0.000
Anxiety symptoms	1.13	0.77–1.64	0.532
Depressive symptoms	1.63	1.07–2.47	0.022

CI, confidence interval.

In contrast, response to PTSD-related flashbacks was not associated with increasing THC:CBD ratio (OR: 1.43; 95% CI: 0.60–3.41; *p* = 0.415). Similarly, there was no association between ratio and response for anorexia (OR: 1.61; 95% CI: 0.70–3.73; *p* = 0.265) or for anxiety symptoms (OR: 1.13; 95% CI: 0.77–1.64; *p* = 0.53).

**FIG. 1.**
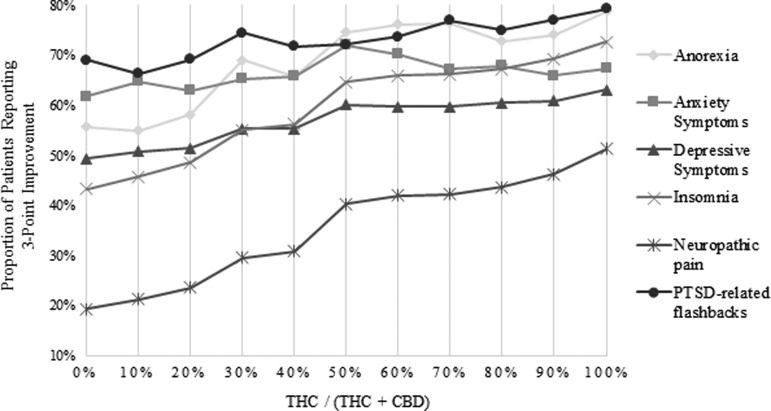
Adjusted response rates (three-point improvement) for six common palliative care symptoms.

Finally, we used each logistic regression model to adjust the proportion of patients who reported a three-point improvement for age, gender, and dose. We grouped THC:CBD ratios in 10 categories, corresponding to the percentage of THC (0–100). These results are displayed in Figure 1.

We looked first at the three symptoms for which an increasing THC:CBD ratio was significantly associated with response (neuropathic pain, insomnia, and depressive symptoms). Of these, the largest relative increase was seen for neuropathic pain (from 19.3% to 51.3%), a greater than twofold change. There were substantial increases for insomnia as well (from 43.3% to 72.3%). The increases for depressive symptoms were more modest, but linear (from 49.4% to 63.1%).

For anxiety symptoms, there was an inverted U-shaped curve that demonstrated increasing response up to a 1:1 ratio (72.0%), and then a decreasing response. For PTSD-related flashbacks, there was a small increase in responses (adjusted proportions 69.0% to 79.3%). There was a larger absolute increase for anorexia (55.8% to 78.8%) but, as for PTSD-related flashbacks and anxiety symptoms, this increase was not statistically significant.

## Discussion

Enthusiasm for medical cannabis is rapidly outstripping the evidence to support its use. Randomized controlled trials are needed to provide guidance to patients, clinicians, and policy makers. However, in the meantime, these results offer useful preliminary guidance for the use for medical cannabis, and can suggest directions for future research. There are four results in particular that may be clinically useful and which should prompt future research.

First, these results underscore the importance of the THC:CBD ratio as an important attribute that patients and clinicians should consider in treatment decisions. This is particularly true for neuropathic pain, insomnia, and depressive symptoms, for which this ratio seems to explain considerable variance in perceived effectiveness. For neuropathic pain in particular, an increase in THC:CBD ratio over the full range (0–100%) more than doubled perceptions of effectiveness.

Second, some symptoms may not be as responsive to increases in the THC:CBD ratio. For PTSD-related flashbacks and anorexia, although adjusted proportions showed a trend toward an increase, this trend was not significant. Indeed, the evidence to support the use of cannabis for PTSD-related symptoms is still very sparse,^11^ although several clinical trials are underway.

And for anxiety symptoms, increases above a ratio of 1:1 were associated with reduced effectiveness. This inverted U-shaped curve seen for anxiety symptoms is not surprising. One well-recognized side effect of THC is anxiety symptoms, sometimes accompanied by paranoia. So it is reasonable that even if THC offers some symptom relief, higher ratios may have the opposite effect.

Two other results are more surprising and warrant further exploration. First, the finding that increasing THC:CBD ratios are associated with greater effectiveness for neuropathic pain is unexpected. There is increasing evidence that CBD offers benefits in the treatment of neuropathic pain, and some suggestion that CBD-only formulations may be effective.^9,12–17^ And yet these results indicate a strong effect of increasing THC:CBD ratio, in a linear manner. It is also noteworthy that the overall improvement rate noted here for neuropathic pain (47%) is higher than that reported in a recent meta-analysis (20%).^18^ Further research is needed to explore the observed differences between these findings in a real-world population and the previous literature.

Second, these results did not indicate that a higher THC:CBD ratio is associated with greater effectiveness in the treatment of anorexia. This is also unexpected, because much of the evidence for the use of cannabis for this symptom comes from studies of either THC-dominant cannabis strains or synthetic THC (dronabinol).^19–21^ Those studies are generally consistent in demonstrating modest effectiveness. Therefore, the results reported here suggest that CBD may also have a role to play in the treatment of anorexia.

Although this study offers some surprises and can make valuable suggestions for future research, there are three limitations that should be considered in interpreting the results reported here. First, this study relied entirely on patients' reports of their own symptoms. We cannot establish, for instance, that these patients actually suffered from clinically diagnosed depression or neuropathic pain. Although this is a significant limitation, it is a limitation that would be expected to introduce noise into potential associations of interest. That is, errors in diagnostic classification would tend to reduce the ability to detect differences in the association between THC:CBD ratio and perceived effectiveness across symptoms. The net effect of diagnostic misclassification would be to produce underestimates of relationships. Therefore, although this study may have failed to detect real associations (e.g., for anorexia or depressive symptoms), one can still be confident that the associations that were identified are genuine.

Second, in this study we had access to very little additional data about patients. For instance, we are unable to determine the etiology for a patient's report of neuropathic pain or anorexia. Even the presence of diagnosis data cannot be used to ascribe etiology with any certainty. Nor could we use additional data such as body mass, functional status, or other medications that may be associated with responsiveness to cannabinoids. These and other limitations could be remedied by future registry studies that link to electronic health records.

Finally, it is important to note that this study may overestimate THC's value because of the placebo effect. Because THC has psychoactive effects, it is easy for patients to be cued by the sensation of euphoria to expect benefits. This limitation is not specific to this sort of observational study, of course. In fact, it is a broad concern about all clinical studies of THC-containing cannabis, which makes true blinding impossible.^22^

Although these limitations are important, this observational study provides important and novel evidence that this approach can offer important insights into use patterns and perceptions of effectiveness. By leveraging large number of patients, using cannabis in their daily routines, large-scale registry studies can provide valuable real-world data. Although such studies cannot replace randomized controlled trials, they can—and should—be an essential part of the growing science of medical cannabis.

## References

[1] Aggarwal SK, Carter GT, Sullivan MD, et al.: Prospectively surveying health-related quality of life and symptom relief in a lot-based sample of medical cannabis-using patients in urban Washington State reveals managed chronic illness and debility. Am J Hosp Palliat Care 2013;30:523–531 22887696 10.1177/1049909112454215

[2] Luba R, Earleywine M, Farmer S, et al.: Cannabis in end-of-life care: Examining attitudes and practices of palliative care providers. J Psychoactive Drugs 2018;50:348–354 29714640 10.1080/02791072.2018.1462543

[3] Mechoulam R: Cannabis—A valuable drug that deserves better treatment. Mayo Clin Proc 2012;87:107–109 22305022 10.1016/j.mayocp.2011.12.002 PMC3498425

[4] Whiting PF, Wolff RF, Deshpande S, et al.: Cannabinoids for medical use: A systematic review and meta-analysis. JAMA 2015;313:2456–2473 26103030 10.1001/jama.2015.6358

[5] Stockings E, Campbell G, Hall WD, et al.: Cannabis and cannabinoids for the treatment of people with chronic noncancer pain conditions: A systematic review and meta-analysis of controlled and observational studies. Pain 2018;159:1932–1954 29847469 10.1097/j.pain.0000000000001293

[6] Medicine NAoSEa: The Health Effects of Cannabis and Cannabinoids: The Current State of Evidence and Recommendations for Research. www.nationalacademies.org/hmd/Reports/2017/health-effects-of-cannabis-and-cannabinoids.aspx (last accessed 127, 2018) 28182367

[7] Martin-Sanchez E, Furukawa TA, Taylor J, et al.: Systematic review and meta-analysis of cannabis treatment for chronic pain. Pain Med 2009;10:1353–1368 19732371 10.1111/j.1526-4637.2009.00703.x

[8] Nugent SM, Morasco BJ, O'Neil ME, et al.: The effects of cannabis among adults with chronic pain and an overview of general harms: A systematic review. Ann Intern Med 2017;167:319–331 28806817 10.7326/M17-0155

[9] Ellis RJ, Toperoff W, Vaida F, et al.: Smoked medicinal cannabis for neuropathic pain in HIV: A randomized, crossover clinical trial. Neuropsychopharmacology 2009;34:672–680 18688212 10.1038/npp.2008.120 PMC3066045

[10] Rog DJ, Nurmikko TJ, Friede T, et al.: Randomized, controlled trial of cannabis-based medicine in central pain in multiple sclerosis. Neurology 2005;65:812–819 16186518 10.1212/01.wnl.0000176753.45410.8b

[11] O'Neil ME, Nugent SM, Morasco BJ, et al.: Benefits and harms of plant-based cannabis for posttraumatic stress disorder: A systematic review. Ann Intern Med 2017;167:332–340 28806794 10.7326/M17-0477

[12] Wilsey B, Marcotte T, Deutsch R, et al.: Low-dose vaporized cannabis significantly improves neuropathic pain. J Pain 2013;14:136–148 23237736 10.1016/j.jpain.2012.10.009 PMC3566631

[13] Ware MA, Wang T, Shapiro S, et al.: Smoked cannabis for chronic neuropathic pain: A randomized controlled trial. CMAJ 2010;182:E694–E701 20805210 10.1503/cmaj.091414 PMC2950205

[14] Wilsey B, Marcotte T, Tsodikov A, et al.: A randomized, placebo-controlled, crossover trial of cannabis cigarettes in neuropathic pain. J Pain 2008;9:506–521 18403272 10.1016/j.jpain.2007.12.010 PMC4968043

[15] Koppel BS, Brust JC, Fife T, et al.: Systematic review: Efficacy and safety of medical marijuana in selected neurologic disorders: Report of the Guideline Development Subcommittee of the American Academy of Neurology. Neurology 2014;82:1556–1563 24778283 10.1212/WNL.0000000000000363 PMC4011465

[16] Hoggart B, Ratcliffe S, Ehler E, et al.: A multicentre, open-label, follow-on study to assess the long-term maintenance of effect, tolerance and safety of THC/CBD oromucosal spray in the management of neuropathic pain. J Neurol 2015;262:27–40 25270679 10.1007/s00415-014-7502-9

[17] Serpell M, Ratcliffe S, Hovorka J, et al.: A double-blind, randomized, placebo-controlled, parallel group study of THC/CBD spray in peripheral neuropathic pain treatment. Eur J Pain 2014;18:999–1012 24420962 10.1002/j.1532-2149.2013.00445.x

[18] Andreae MH, Carter GM, Shaparin N, et al.: Inhaled cannabis for chronic neuropathic pain: A meta-analysis of individual patient data. J Pain 2015;16:1221–1232 26362106 10.1016/j.jpain.2015.07.009 PMC4666747

[19] Dejesus E, Rodwick BM, Bowers D, et al.: Use of dronabinol improves appetite and reverses weight loss in HIV/AIDS-infected patients. J Int Assoc Physicians AIDS Care (Chic) 2007;6:95–100 17538000 10.1177/1545109707300157

[20] Beal JE, Olson R, Laubenstein L, et al.: Dronabinol as a treatment for anorexia associated with weight loss in patients with AIDS. J Pain Symptom Manage 1995;10:89–97 7730690 10.1016/0885-3924(94)00117-4

[21] Jatoi A, Windschilt HE, Loprinzi CL, et al.: Dronabinol versus megestrol acetate versus combination therapy for cancer-associated anorexia: A North Central Cancer Treatment Group study. J Clin Oncol 2002;20:567–573 11786587 10.1200/JCO.2002.20.2.567

[22] Casarett D: The achilles heel of medical cannabis research-inadequate blinding of placebo-controlled trials. JAMA Intern Med 2018;178:9–10 29159413 10.1001/jamainternmed.2017.5308

